# Improving regional influenza surveillance through a combination of automated outbreak detection methods: the 2015/16 season in France

**DOI:** 10.2807/1560-7917.ES.2017.22.32.30593

**Published:** 2017-08-10

**Authors:** Camille Pelat, Isabelle Bonmarin, Marc Ruello, Anne Fouillet, Céline Caserio-Schönemann, Daniel Levy-Bruhl, Yann Le Strat, the Regional Influenza study group

**Affiliations:** 1 Santé publique France, French national public health agency. Saint-Maurice, France; 2 Members of the regional influenza study group are mentioned at the end of the article

**Keywords:** influenza, influenza-like illness, ILI, automated surveillance, syndromic surveillance, sentinel surveillance, statistics

## Abstract

The 2014/15 influenza epidemic caused a work overload for healthcare facilities in France. The French national public health agency announced the start of the epidemic – based on indicators aggregated at the national level – too late for many hospitals to prepare. It was therefore decided to improve the influenza alert procedure through (i) the introduction of a pre-epidemic alert level to better anticipate future outbreaks, (ii) the regionalisation of surveillance so that healthcare structures can be informed of the arrival of epidemics in their region, (iii) the standardised use of data sources and statistical methods across regions. A web application was developed to deliver statistical results of three outbreak detection methods applied to three surveillance data sources: emergency departments, emergency general practitioners and sentinel general practitioners. This application was used throughout the 2015/16 influenza season by the epidemiologists of the headquarters and regional units of the French national public health agency. It allowed them to signal the first influenza epidemic alert in week 2016-W03, in Brittany, with 11 other regions in pre-epidemic alert. This application received positive feedback from users and was pivotal for coordinating surveillance across the agency’s regional units.

## Introduction

The influenza A(H3N2) strain that circulated in France during the winter 2014/15 caused an overload of patients in hospitals’ emergency departments (ED), with more than 30,000 visits, and in intensive care units, with at least 1,600 severe cases [1]. The hospitalisation rate after ED visit was high (11% vs 6–9% usually since 2009), especially among elderly people (47%).

Santé publique France, the French national public health agency, announced the start of the epidemic at the national level when the national incidence rate of influenza-like illness (ILI) cases who consulted general practitioners belonging to a surveillance sentinel network crossed the epidemic threshold (determined using a periodic regression model) [2,3]. However, this was too late for many hospitals to prepare.

There was no communication about the epidemic’s imminent arrival in different administrative regions of France, although it is known that the start of the epidemic is not the same in all regions. Weekly influenza reports were produced in the national public health agency’s regional units, based on a variety of data and statistical methods, with no inter-regional comparison.

The national public health agency’s influenza alert procedure was subsequently reorganised to improve timeliness of alerts by implementing the following surveillance system features: (i) A pre-epidemic alert level was introduced to better anticipate the rise in ED visits and bed occupancy rates needed during epidemics; (ii) Epidemiologists in the national public health agency’s regional units were delegated the responsibility of determining the influenza alert level in the 22 administrative regions of metropolitan France (the part of France in Europe) every week and informing regional health authorities of the arrival of the epidemic in their region; (iii) The determination of the alert levels was aided by the standardised use of data sources and statistical outbreak detection methods.

In order to implement these three measures and help epidemiologists make better informed decisions about influenza alert levels, we developed a web application called MASS (Module for the Analysis of SurSaUD and Sentinelles’ data). MASS compiles data from two surveillance systems in near real-time, computes statistical alarm levels and proposes data visualisations that highlight inter-regional comparisons. Although influenza was our primary target, our goal was to create a tool suitable for the monitoring of various health indicators, in particular seasonal infections. Accordingly, 11 other so-called ‘syndromic groups’ were also included in this project: five with (most probable) infectious origin and winter peaks (acute lower respiratory infections, bronchitis, bronchiolitis, gastroenteritis and pulmonary diseases), asthma, isolated fever, meningitis, faintness, food-poisoning and wild mushroom poisoning.

In this paper, we present MASS, focusing on influenza. We describe its role within Santé publique France’s new surveillance workflow during the 2015/16 influenza season in metropolitan France and discuss its added value.

## Methods

### Data sources

#### SurSaUD: Emergency departments and emergency general practitioners

In 2004, Santé publique France set up the syndromic surveillance system SurSaUD, based on data from ED attendance, all-cause mortality and, from 2006 onwards, also data from emergency general practitioner (GP) visits [4,5]. Below we describe the SurSaUD data used in MASS. Mortality data were not used in MASS since these data usually lag behind morbidity data and therefore are not helpful in detecting influenza epidemic periods. Also, only the data after 1 January 2010 were used in MASS, as this corresponded to the time when the system reached a sufficient and stable coverage of the French population.

ED data are collected from computerised medical records completed during consultations in the hospitals involved in the Organisation of the coordinated surveillance of ED network (organisation de la surveillance coordonnée des urgences (OSCOUR)) [6]. This network grew from 23 hospitals in 2004 to 650 in 2016, capturing ca 88% of all ED visits and covering all French metropolitan administrative regions.

The participating hospitals transmit data to Santé publique France every day before 06:30. The files sent on a given day contain data on the patients seen during the previous 7 days. Most of the patients’ information is transmitted the day following the visit.

The transmitted data contain among other information: the diagnosis code according to the 10th international classification of disease (ICD-10), the consultation date and the patient’s age.

SOS Médecins is a federation of private emergency GP associations. In 2006 it started participating in SurSaUD with 24 associations, reaching 61 in 2016 (out of a total of 62, i.e. covering ca 95% of emergency GP consultations). All 22 metropolitan French regions have at least one participating association. When a patient calls an association to organise a home visit, their age and primary health complaint are registered. After consultation, the doctor registers the diagnosis code on a mobile application, using a specific thesaurus. Every morning, participating associations send their data to a national platform which synthesises all data into a single file which is sent to our agency at 06:00. The transmitted information contains among other details: the SOS Médecins diagnosis code, the consultation date and the patient’s age.

The received data are inserted in a database. An aggregated table is automatically updated with the daily number of visits by reporting unit (hospital ED and GP association), age group (several partitions) and syndromic group. More specifically, for ED data, the syndromic group ILI is composed of ICD-10 codes J09, J10 and J11. For SOS Médecins data, it comprises the three following codes from their own thesaurus: ‘ILI or suspected influenza’, ‘influenza confirmed by test’ and ‘pandemic influenza confirmed by test’.

Every morning, MASS extracts from this aggregated table the ILI data of the last 7 days corresponding to the following age groups: all ages, < 2, < 5, < 15, 2–14, 5–14, ≥ 15, 15–64, 15–74, ≥ 65, 65–74, ≥ 75, ≥ 85 years. MASS calculates, for ED and SOS Médecins data, the proportion of ILI cases among the total number of visits with a valid diagnostic code, which comprise all diagnostic types: diseases, accidents, etc. The proportion of ILI is calculated by age group, geographical area (France, metropolitan France and administrative regions), and by day, week and month. This indicator is preferred over the raw number of ILI cases, since the latter is sensitive to changes in the number of reporting units and changes in the proportion of coded diagnoses.

#### Sentinel general practitioners

*‘*Sentinelles’ is an electronic surveillance system set up in 1984, which collects individual data about consultations in general practice in the general population, using a network of ca 455 volunteer sentinel GPs (in 2015). They represent 0.76% of all GPs in metropolitan France, with wide variations across administrative regions, (from 0.3% in Picardy to 7.1% in Corsica) [7]. The definition of ILI used by Sentinelles is: sudden onset of fever > 39°C (> 102°F) with respiratory signs and myalgia. Sentinelles estimates the weekly incidence rates of ILI consultation in general practice for 100,000 inhabitants using a Horvitz–Thompson estimator in which the sampling weights of the ILI cases depend on the number of GPs participating in the surveillance, the total number of GPs, and the number of inhabitants in each area of interest, each week [8]. This implies that incidence rate estimates can be compared in space and time despite differences in GPs’ participation, overall numbers of GPs, and population size. However, the confidence interval of each weekly estimate depends on the number of participating GPs in the area in that week.

Sentinelles has developed an R package to make the estimates of all-age incidence rates and their confidence intervals accessible to external applications such as MASS. Until February 2017, when a new version of the package was released, the incidence rates by age group were not accessible through this package, and thus were not included in MASS.

The Sentinelles ILI data included in MASS are the all-age incidence rates of ILI consultation in general practice and their confidence intervals, from week 2010-W01 onwards, for the whole of metropolitan France and for all administrative regions. Every Monday at 14:00, Sentinelles releases preliminary estimates of incidence rates for the previous week. Every Tuesday at 10:30, Sentinelles updates these estimates, based on the data reported on Monday. Besides, because the sentinel GPs can report case data up to 12 days after consultation, the incidence rate estimates are updated during the 2 weeks following their initial release. Consequently, to provide up-to-date data in MASS, the all-age incidence rates for the 3 preceding weeks and their confidence intervals were downloaded from the Sentinelles database every Monday at 14:00 and every Tuesday at 10:30.

#### Comparison of case characteristics

Common case information reported by all data providers included age, sex and whether the cases were hospitalised following their visit to the ED or encouraged to do register at a hospital by its Sentinelles or SOS Médecins GP. These pieces of information were not available in real time from Sentinelles and were requested retrospectively at the end of the 2015/16 season.

We compared the case distribution by age groups, sex and hospitalisation status to understand the specificities of each data source, as this knowledge could be useful for future refinement of MASS. The 95% confidence interval of the proportion of cases in each group was calculated using 1 − (*p_s_* × *q_s_* × *r_s,v_*) as a finite population correction factor, *p_s_* being the coverage of the data source *s*, *q_s_* the proportion of reported cases that have a valid diagnostic code in data source *s*, and  *r_s,v_* the proportion of non-missing values for the variable *v* in data source *s* [9].

### Statistical analysis

The weekly time series of ILI proportions (SurSaUD) and incidence rates (Sentinelles), for all ages, were analysed with statistical outbreak detection methods, separately for each geographical area. To allow comparisons, each method is tuned with the same set of parameters for all data sources and areas. We briefly describe the methods used below. All statistical analyses were performed with R [10].

#### Periodic regression

This method has been used by many authors to predict the baseline level of an epidemiological time series in the absence of influenza, and to flag values significantly above this baseline as a possible epidemic [3,11,12]. We modelled the baseline level with a multivariable linear regression model using the following equation:

E(y_w_)=α_0_ + α_1_w + γ_1_cos(2πw/52.17) + δ_1_sin(2πw/52.17) + γ_2_cos(4πw/52.17) + δ_2_sin(4πw/52.17),

where *y_w_* is, for week *w*, the proportion of visits for ILI among all visits with a valid diagnosis code (for ED and SOS Médecins data), or the incidence rate for 100,000 inhabitants (for Sentinelles data).

The predicted baseline level ${\hat{y}}_{w}$ in a given week *w* was obtained by fitting the model to the observations of the past 5 years (i.e. 261 weeks: from week *w* − 261 to week *w* − 1) that were below their 85% percentile. This trimming was an attempt to ensure that the dataset used to model the baseline level was free of influenza. An observation was flagged as possibly indicative of an epidemic if it exceeded the upper limit of the 95% bilateral prediction interval of the predicted baseline.

#### Robust periodic regression

Robust periodic regression resembles periodic regression but does not require trimming [13]. Instead, an iterative fitting algorithm allows small weights to be attributed to the highest values of the dataset (those which indicate a possible influenza epidemic). We used the same model equation and the same model training period (week *w* − 261 to week *w* − 1) as for the periodic regression method. Similarly, an observation was flagged as possibly indicative of an epidemic if it exceeded the upper limit of the 95% prediction interval of the predicted baseline. We employed the rlm function of the R package MASS [14].

#### Hidden Markov model

We used a two-state hidden Markov model to differentiate between epidemic and non-epidemic weeks [15-18]. In our model, under state 1 (non-epidemic state), the observations were normally distributed with mean µ_1_ and variance σ_1_^2^; under state 2 (epidemic state), the observations were normally distributed with mean µ_2_ and variance σ_2_^2^.

Before fitting the model, the missing values occurring between two observations were imputed from the mean of these two observations. The model was then applied sequentially to infer the state of each observation based on the given observation plus the preceding 260 observations. This subset of weeks was first used to estimate µ_1_ (respectively µ_2_) as the mean of the observations below (upper) the 80% percentile, and σ_1_^2^ (σ_2_^2^) as the variance of the observations below (upper) the 80% percentile. The state of the observation of interest was inferred by fitting the model with maximum likelihood techniques, using the R package HiddenMarkov [19].

#### Synthesis of statistical alarms

An influenza alarm level was created for every week in each area by combining the results of the detection methods as follows:

$$\text{alarm level}\;\{\begin{array}{c} \text{non-epidemic if}\;p<0.4\\ \text{pre/post-epidemic if}\;0.4\leq p<1\\ \text{epidemic if }p=1 \end{array}$$

where *p* = *n_a_/n* is based on *n*, the number of statistical results (= 9 when the three detection methods provided a result for all three data sources, and < 9 if one or more data sources are missing or if one or more of the methods fail) and *n_a_* the number of epidemic alarms (between 0 and *n*).

The cut-off values were determined from historical data, under the following two constraints: They should (i) avoid issuing non-epidemic or post-epidemic alarm levels in the ascending phase of the epidemic and (ii) ensure that pre-epidemic alarms were issued.

### Web application

**Figure 1:**
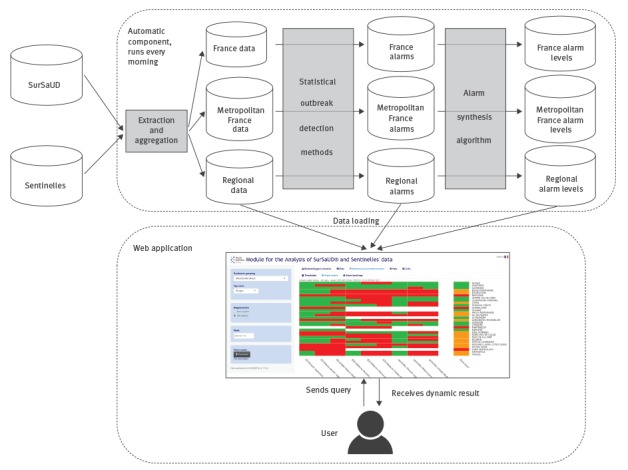
Flow diagram of the web application MASS (Module for the Analysis of SurSaUD and Sentinelles’ data), France, 2015/16

The development of MASS started in June 2015, based on the shiny R package [20]. At the end of July, a video demonstrating the use of the prototype was sent to 31 epidemiologists, in the regional units and at the national level, along with a web questionnaire asking for their opinions and suggestions for improvements. The answers influenced the development of MASS. The application was launched in December 2015, and refined at different moments during the following months. MASS can be accessed from within the national public health agency headquarters or from regional units, in the latter case via a secured network. The schematic process is illustrated in Figure 1.

**Figure 2:**
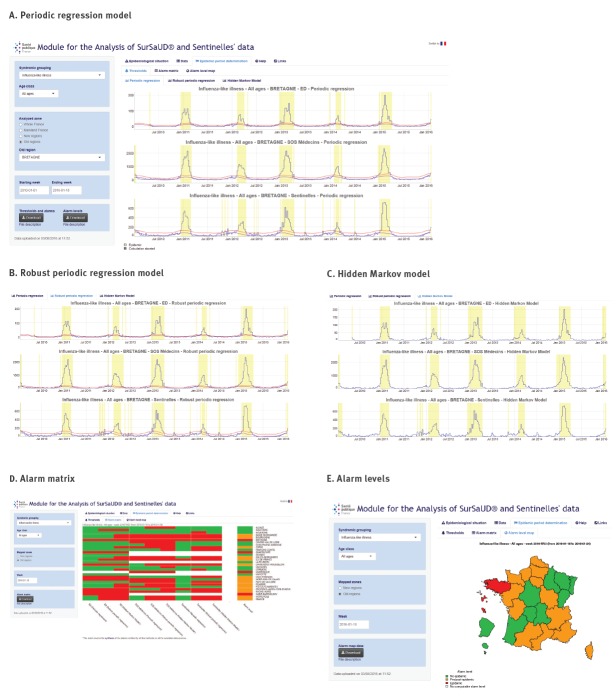
Determination of influenza epidemic periods with the web application MASS (Module for the Analysis of SurSaUD and Sentinelles’ data), France, 2015/16

The interface is organised in tab panels. Dropdown menus and radio buttons on the left hand side can be used to filter the data to display by syndromic group, data source, age group, area, date and time step. In particular, the ‘Data’ panel offers dynamic charts, tables, gauges and maps, and the ‘Detection of epidemic periods’ panel presents statistical alarms and alarm levels (Figure 2). The R code in MASS is available on GitHub (https://github.com/cpelat/MASS) and a demonstration is available at https://cpelat.shinyapps.io/mass/.

Tab panels present the results of three statistical methods of outbreak detection applied to the three data sources included in MASS. The ‘Thresholds’ panel displays the predicted baseline (blue), epidemic threshold (red) and epidemic alarms (yellow bars) obtained with A. the periodic regression model, B. the robust periodic regression model and C. the hidden Markov model, on the three data sources. D. The alarm matrix presents the results of the outbreak detection methods side by side, along with the alarm level obtained by their synthesis. E. The alarm levels are displayed on a map, where France is partitioned along old administrative regions (those in use before 2016), i.e. 22 in metropolitan France and five overseas territories.

### Alert procedure

Every week during the 2015/16 influenza surveillance period, the epidemiologists in the national public health agency’s regional units were asked to choose an alert level for the region(s) they were in charge of: non-epidemic, pre-/post-epidemic or epidemic. The epidemiologists based their analysis on the MASS alarm levels and on other relevant information such as virological data or local surveillance systems, including the surveillance of clusters of acute respiratory infections in nursing homes. Their decision about which level to report could also take into account the precision of each data source in their region and the alarm level in neighbouring regions.

Every Tuesday morning, the regional epidemiologists entered their chosen alert level(s) in a dedicated web questionnaire and, if appropriate, the reason why they proposed an alert level different from the MASS alarm level. The proposed alert levels were reviewed collectively at the national public health agency’s headquarters every Tuesday at noon and discussed in the weekly conference call that was held every Tuesday at 16:00 with the national surveillance partners (e.g. virologists from the influenza reference centres, epidemiologists and virologists from the Sentinelles network). The validated alert levels were published on Wednesdays in the weekly national influenza report posted on the agency website. The regional units of the national public health agency informed their regional health agency and communicated alert levels through their regional epidemiological reports.

## Results

### The 2015/16 influenza epidemic

**Figure 3:**
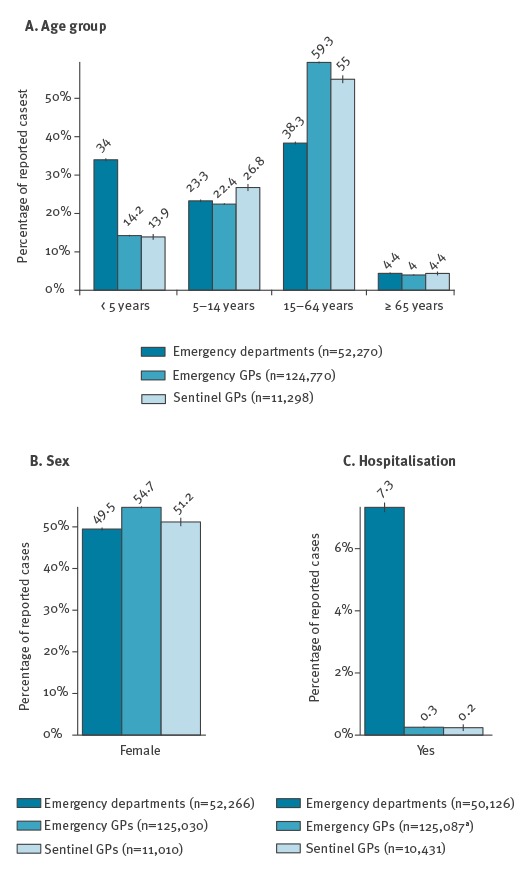
Distribution of influenza-like illness cases reported by three data source providers used in the web application MASS (Module for the Analysis of SurSaUD and Sentinelles’ data), France, 2015/16 influenza surveillance period (week 2015-W50 to 2016-W17)

The new procedure for influenza alert in metropolitan France was implemented from week 2015-W50 to 2016-W17. Among the 2,144 viruses isolated in that season in ambulatory medicine through random sampling of ILI cases, 70% were influenza B viruses (lineage B/Victoria), and 27% belonged to the subtype A(H1N1)pdm09 [21]. During this period, participating ED staff reported 52,271 ILI cases, SOS Médecins’ associations 125,087 cases and Sentinelles GPs 11,298 cases. Figure 3 presents the distribution of ILI cases by age group, sex and whether hospitalisation followed the visit or was encouraged.

GP: general practitioner.

The vertical black lines represent the 95% confidence intervals. The numbers in brackets represent the cases without missing value in each data source, for each variable of interest. Data are presented by A. age group, B. percentage of female cases and C. percentage of cases hospitalised following their visit to the emergency department or directed to the hospital by their GP.

^a^ The variable recording the hospitalisation status in the Emergency GPs’ data (SOS Médecins) is coded ‘H’ (case encouraged to go to hospital) or blank. In other words, cases with missing hospitalisation status cannot be distinguished from cases not directed to the hospital, thus the proportion of cases directed to the hospital may be underestimated.

**Figure 4:**
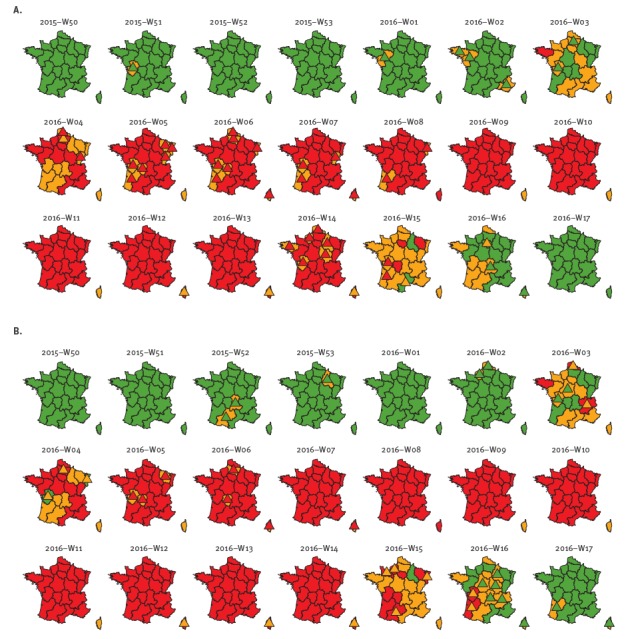
Weekly influenza alarm levels (generated by statistical methods) and alert levels (resulting from epidemiological validation), 2015/16 surveillance period (week 2015-W50 to 2016-W17)

MASS was used every week throughout this period by the epidemiologists involved in influenza surveillance at the national public health agency’s regional and national levels. The influenza alert levels published in the weekly reports are shown in Figure 4, panel A, along with the MASS alarm levels, to highlight the discrepancies between them. The first region to signal an epidemic was Brittany, in week 2016-W03, with 11 other regions at a pre-epidemic alert level. All regions were at the epidemic alert level within the subsequent 2 weeks (except Corsica, 3 weeks). MASS emitted the first epidemic alarm level for the area of metropolitan France in week 2016-W04, and the epidemic was declared at the national level in the same week.

The background colours represent the alarm levels. Green: non-epidemic, orange: pre-/post-epidemic, red: epidemic. The alert levels are represented by coloured triangles where and when they differ from the alarm levels. A. The alarm levels are those produced by the web application MASS, based on three data sources and three outbreak detection methods. B. Alternative calculation of the alarm levels, based on only two data sources (emergency departments and emergency general practitioners) and two methods (periodic regression and robust periodic regression models).

All French metropolitan regions except Corsica remained at epidemic alert level until week 2016-W14, although MASS emitted post-epidemic alarms levels for six regions in that same week. Most regions then transited through the post-epidemic alert level to the non-epidemic level, and the epidemic was declared over in all regions of metropolitan France in week 2016-W17. At the level of metropolitan France, the post-epidemic alarm level was signalled and epidemiologically validated in week 2016-W15. The non-epidemic alarm level was signalled in week 2016-W16 and the end of the national epidemic was declared.

### Performance

The alarm level was based on nine statistical results for 381 of the 462 analysed region-weeks (22 regions x 21 weeks), and on eight statistical results for the other 81 region-weeks because of failures adjusting the robust periodic regression model to the Sentinelles time series in four regions: Picardy (21 failures over the 21 weeks), Burgundy (21 failures), Upper Normandy (21 failures) and Lower Normandy (18 failures). In these regions, Sentinelles ILI incidence rate was zero for more than half of the weeks (vs less than half for the other regions).

Discrepancies between the MASS alarm levels and the epidemiologists’ decisions about levels accounted for less than 10% of the decisions (44/462): nine pre-/post-epidemic alarm levels from MASS were transformed into non-epidemic alert levels, 29 into epidemic alert levels; four epidemic and two non-epidemic alarm levels from MASS were transformed into pre-/post-epidemic alert levels.

### Sensitivity analysis

We recalculated the regional alarm levels for the influenza surveillance period 2015/16 using all possible subsets of data sources and outbreak detection methods, and counted the discrepancies with the epidemiologists’ decisions (Table). The best alternative, generating only 36 discrepancies, was based on data from ED and SOS Médecins only with the periodic regression and robust periodic regression models (Figure 4, panel B).

**Table:** Number of discrepancies between epidemiologists’ decisions concerning influenza alert level and the alarm level calculated using different subsets of data sources and outbreak detection methods, among the 462 region-weeks of the surveillance period (22 regions x 21 weeks).

Data source(s)	1 method			2 methods			3 methods
	PR	Robust PR	HMM	PR and robust PR	PR and HMM	Robust PR and HMM	PR and robust PR and HMM
ED	NC	NC	NC	NC	NC	NC	65
SOS Médecins	NC	NC	NC	NC	NC	NC	59
Sentinelles	NC	NC	NC	NC	NC	NC	86^a^
ED and SOS Médecins	NC	NC	NC	36	44	76	39
ED and Sentinelles	NC	NC	NC	62 ^a^	69	72 ^a^	67 ^a^
SOS Médecins and Sentinelles	NC	NC	NC	64 ^a^	69	76 ^a^	74 ^a^
ED and SOS Médecins and Sentinelles	56	55^a^	63	45 ^a^	47	57 ^a^	44 ^a^

ED: emergency department; HMM: Hidden Markov Model; PR: periodic regression model.

NC*:* the alarm level was not computed for those combinations, which provide at most one or two results to combine.

^a^ The robust PR model applied on the Sentinelles time series failed to provide a result for 81 region-weeks. In those 81 occasions, the alarm level was based on n − 1 statistical results (n being the number of statistical methods multiplied with the number of data sources).

Periodic regression and robust periodic regression models outperformed Hidden Markov Models, both when used alone and in combinations. We explored the cause for discrepancy for each method when used alone for all three data sources. For the periodic regression model, 51 of 56 discrepancies were due to the epidemiologists choosing an alert level higher than the alarm level. In contrast, most of the discrepancies between the epidemiologists’ alert level and the alarm levels produced by the robust periodic regression model and the Hidden Markov were the result of epidemiologists choosing a lower alert level (41/55 and 51/63 discrepancies, respectively).

## Discussion

### Added value

For the first time, our agency was able to announce the start of the influenza epidemic at the regional level, based on a standardised procedure across regions, coordinated at the national level. The lag between the first and last regions to enter the epidemic phase was 3 weeks. This asynchronicity and the fact that public health decisions concerning influenza are taken by regional authorities are a reminder that monitoring influenza at the regional level is essential.

The regional health agencies were informed of the advent of the pre-epidemic phase, then of the epidemic phase, which enabled hospitals to progressively adapt the healthcare provision if needed. Overall, MASS proved useful for local health service planning, even though the impact on healthcare services of the influenza epidemic in 2015/16 was less marked than in 2014/15.

### User feedback

User feedback on the new influenza alert procedure was positive. Epidemiologists appreciated the increased inter-regional and national–regional coordination, where MASS proved pivotal. The fact that MASS was developed in close collaboration with its end users probably contributed to its rapid integration into routine surveillance activities. From our experience, the shiny R package is well suited for building specific reporting tools that need frequent adaptation to users’ needs.

### Agreement between MASS and epidemiologists

The level of agreement between MASS alarm levels and epidemiological decisions regarding alert levels was high during the surveillance period. The changes made by the epidemiologists aimed at correcting levels that seemed unlikely or at following the expected alert level sequence.

With respect to the first point, one example is where MASS signalled an early pre-epidemic alarm level for Poitou-Charentes in week 2015-W51, when all the other regions were non-epidemic. The epidemiologists maintained the non-epidemic alert level. History proved them right as MASS classified the subsequent weeks as non-epidemic. Similarly, MASS signalled a post-epidemic alarm level in four regions after only 1–3 epidemic weeks but the epidemiologists maintained the epidemic alert level because such a short epidemic was unlikely. Here again, history proved them right as MASS classified the subsequent weeks as epidemic.

With respect to the latter point, the expected alert level sequence was: ‘non-epidemic/pre-epidemic/epidemic/post-epidemic/non-epidemic’. For example, in the Ile-de-France and Languedoc-Roussillon regions, a non-epidemic MASS alarm level directly followed the epidemic level and was transformed by epidemiologists into a post-epidemic level. However, in some instances (e.g. week 2016-W02 in Brittany), the epidemiologists preferred not to validate the pre-epidemic alarm level in the MASS sequence, maybe because of doubts about the application’s functioning or about the precision of some data sources at that time. As a result, Brittany transited directly from the non-epidemic alert level (week 2016-W02) to the epidemic alert level (week 2016-W03).

As pre- (and post-) epidemic phases are important for health service preparedness, we evaluated how well MASS and the epidemiologists detected them: MASS bypassed the pre-epidemic phase in one region (vs three for the epidemiologists) and the post-epidemic phase in three regions (vs two for the epidemiologists).

### Limitations and perspectives

Although MASS produced valuable results for routine work, areas of improvement were identified. Firstly, for the sake of simplicity, all data sources and detection methods shared the same weight in the construction of MASS alarm levels: e.g. three alarms on the same data source are equivalent to one alarm for each source. Alternative criteria could be considered here, for example the number of sources with at least one alarm. Besides, in a sensitivity analysis, the alarm level that was closest to the epidemiologists’ decisions relied only on two data sources and two methods. This alternative construction could be considered for future seasons, although it was decided to keep the current construction for the 2016/17 season. Furthermore, the different data sources are associated with different levels of precision, since the number of ILI cases reported by SOS Médecins’ GPs is twice that reported by ED and 10 times that reported by Sentinelles GPs. They also serve different case populations in terms of ILI definition, age distribution (with a higher proportion of children younger than 5 years in ED data and of young active people in SOS Médecins’ data) and severity of symptoms (hospitalisation after visit being much more common in ED), while the sex ratio is ca 1:1 in all surveillance systems. All these criteria are considered by the epidemiologists when making their decision but are not yet included in the automatic construction of MASS alarm levels. This could constitute a possible evolution of the tool, although recent analyses indicate that the dynamics of most influenza epidemics vary little between age groups [22]. In theory, a patient could be reported in the three detection systems, and in multiple weeks, but this is unlikely. As the data were anonymised before transmission, we had no means to correct for that possibility.

Future developments include the integration of virological data in MASS. 

Finally, several countries have been using an influenza epidemic threshold for years [23,24], yet the best data source and the best outbreak detection method to use are still a matter of discussion [25]. We shall continue to look for the combination of data sources and methods that produces the most relevant results for our needs.

## Notes

**The Regional Influenza study group:** At Santé publique France, French national public health agency, Saint-Maurice, France, Regional units (Cire): Olivier Retel (Cire Bourgogne-Franche-Comté), Bruno Hubert (Cire Pays de la Loire), Franck Golliot (Cire Occitanie), Lisa King (Cire Bretagne), Ibrahim Mounchetrou Njoya (Cire Ile-de-France), Christine Saura (Cire Auvergne-Rhône-Alpes), Laurent Filleul (Cire Océan Indien).
